# Sexual Maturity Promotes Yolk Precursor Synthesis and Follicle Development in Hens via Liver-Blood-Ovary Signal Axis

**DOI:** 10.3390/ani10122348

**Published:** 2020-12-09

**Authors:** Zhifu Cui, Felix Kwame Amevor, Qian Feng, Xincheng Kang, Weizhen Song, Qing Zhu, Yan Wang, Diyan Li, Xiaoling Zhao

**Affiliations:** Farm Animal Genetic Resources Exploration and Innovation Key Laboratory of Sichuan Province, Sichuan Agricultural University, Chengdu 611130, China; 2018102013@stu.sicau.edu.cn (Z.C.); amevorfelix@gmail.com (F.K.A.); 13055564829@163.com (Q.F.); kangxincheng@stu.sicau.edu.cn (X.K.); 2019302063@stu.sicau.edu.cn (W.S.); zhuqingsicau@163.com (Q.Z.); as519723614@163.com (Y.W.); diyanli@sicau.edu.cn (D.L.)

**Keywords:** yolk precursor synthesis, hormones, lipid metabolism, liver-blood-ovary signal axis

## Abstract

**Simple Summary:**

The current work evaluates the liver-blood-ovary signal axis in relation to age changes involved in regulating egg yolk precursor synthesis in chickens. In this study, we observe the morphology and histology of the liver and ovary and determine the serum biochemical parameters and the expression abundance of the critical genes from the age of 90 days (d90) to d153. The mRNA and protein expressions of estrogen receptor-alpha (ER-α) and E_2_ levels in the liver and serum, respectively, showed similar changes. Moreover, with reference to an increased serum E_2_ level, the mRNA expression of genes related to yolk precursor synthesis (very low density apolipoprotein-II (ApoVLDL-II) and vitellogenin-II (VTG-II)), lipogenesis (fatty acid synthase (FAS)), and lipid transport (microsomal triglyceride transport protein (MTTP)) in the liver showed up-regulation.

**Abstract:**

Several reproductive hormones were reported to be involved in regulating egg yolk precursor synthesis in chickens; however, the mechanism that shows how the liver-blood-ovary signal axis works in relation to age changes has not been reported yet. Therefore, in this study, we observe the morphology and histology of the liver and ovary and determine the serum biochemical parameters and the expression abundance of the critical genes from d90 to 153. Results show that the body weight and liver weight were significantly increased from d132, while the ovary weight increased from d139. Aside from the increase in weight, other distinct changes such as the liver color and an increased deposition of large amounts of yolk precursors into the ovarian follicles were observed. On d139, we observed small fatty vacuoles in the hepatocytes. The results of serum biochemical parameters showed a significant increase in the estradiol (E_2_) level, first on d125, and then it reached its peak on d132. Meanwhile, the levels of follicle-stimulating hormone (FSH) increased initially and then remained at a high level from d146 to d153, while the levels of luteinizing hormone (LH) increased significantly on d132 and reached the top level on d153. Moreover, the levels of lecithin (LEC), vitellogenin (VTG), very low density lipoprotein y (VLDLy), triglyceride (TG), and total cholesterol (TC) were significantly increased at d125 and were close from d146 to d153. The mRNA and protein expression of estrogen receptor-alpha (ER-α) and E_2_ levels in the liver and serum, respectively, showed similar changes. Moreover, with reference to an increase in serum E_2_ level, the mRNA expression of genes related to yolk precursor synthesis (very low density apolipoprotein-II, ApoVLDL-II) and vitellogenin-II (VTG-II), lipogenesis (fatty acid synthase, FAS), and lipid transport (microsomal triglyceride transport protein, MTTP) in the liver showed up-regulation. These results suggest that the correlation between liver-blood-ovary alliances regulate the transport and exchange of synthetic substances to ensure synchronous development and functional coordination between the liver and ovary. We also found that E_2_ is an activator that is regulated by FSH, which induces histological and functional changes in the hepatocytes through the ER-α pathway.

## 1. Introduction

The poultry egg is an important nutritional source that contains rich proteins, lipids, vitamins, and minerals [1,2]. Its yolk had abundant energy to ensure embryonic development [3]. The major constituents of the egg yolk are proteins and lipids, which are present mainly in the form of lipoproteins and have several beneficial functions. Egg yolk lipoproteins have been found to possess numerous nutritional and health benefits, such as antimicrobial immunoglobulin Y [4,5,6], antioxidant properties [7,8], and the provision of the required nutrients for infant brain development [9,10,11].

The liver is the largest gland in the chicken and plays vital roles in fat metabolism [12,13] and yolk precursor synthesis [14]. During egg production, the chicken’s liver synthesizes apolipoprotein and vitellogenin (VTG), which are the main components of the egg yolk proteins [15]. Studies indicated that estrogen induced yolk protein synthesis in hens [16] and also increased the synthesis of hepatic apolipoprotein B (apoB) [17]. After the synthesis of these yolk precursors in the liver, APoB is packaged into very low density lipoprotein (VLDL) particles for transportation while VTG is released into the blood to be transported into the oocyte [18]. VLDL and VTG are the key yolk precursors to transport and deposit additional nutrients into the ovary. VTG, as a glycophospholipoprotein, is responsible for supplying glucose, phosphorus, and fat and also binds metal ions such as calcium, zinc, and iron and transports them into the oocyte [19]. 

At the onset of ovulation, estrogen transforms virtually all hepatocytic lipoprotein produced from generic VLDL to VLDLy (yolk targeted) [20]. Triglyceride (TG), lecithin (LEC), and total cholesterol (TC) synthesized by the hepatocytes are mainly transported in the form of VLDLy, which is the only substance that can reach the growing oocyte successfully to form a yolk [21]. ApoVLDL-II is a vital apolipoprotein in the layer chicken and is responsible for assembling and transporting VLDLy. Moreover, the genes encoding ApoVLDL-II and VTG are associated with egg production in chickens [18]. VLDLy enters the growing oocyte in the form of intact lipoprotein particles that are ingested by a concave vesicle on the oocyte membrane, thereby achieving deposition in the follicle [22]. The VLDLy pathway for apolipoprotein provides sufficient energy requirements for follicle development. The main key for ovarian follicle development is attributed to the successful transportation of large amount of nutrients to the oocytes by VLDLy and VTG [23]. 

Sexual maturity is mainly dependent on the regulation of the hypothalamus-pituitary-gonadal (HPG) axis, and factors such as nutrition and light also influence sexual maturity by regulating the hormone levels in the reproductive axis. Although previous studies have shown that egg yolk synthesis and deposition in hens is regulated by diverse hormones [24,25], the age-related changes of liver and ovary and the actual regulatory mechanism are not known. Therefore, in the present study, we focus on the age-related changes in the liver-blood-ovary signal axis until the hen laid its first egg, the morphology and histology of the liver and ovary, serum biochemical parameters, and the expression of critical genes. Our results will expand the data and knowledge on the regulatory mechanism of yolk formation in the chicken.

## 2. Materials and Methods

### 2.1. Animals

Three hundred and six Rohman layers were fed in the poultry farm of Sichuan Agricultural University (Ya’an, Sichuan Province, China) and each bird was raised in a single cage (size: 500 mm × 400 mm × 370 mm). The housing temperature and relative humidity was 20 ± 3 °C and 65–75%, respectively. Light was provided for 8 h (8L: 16D, 5 Lux light intensity) from week 8 to 17, increased by 1 h per week from week 18 to 23 (10–15 Lux light intensity), and then maintained at 14 h thereafter. A corn-soy diet, in pellet form, was provided for the chickens from week 9 to16 and it consisted of 14.5% CP, 0.9% Ca, 0.58% P, and 2800 kcal of ME/kg, and then from week 17 to 23 and thereafter, the composition was 17.5% CP, 2% Ca, 0.65% P, and 2800 kcal of ME/kg. Water was provided ad libitum. The animal experiments were approved by the Institutional Animal Care and Use Committee of Sichuan Agricultural University (Certification No. YCS-B2018102013). All experiments were conducted in accordance with the Sichuan Agricultural University (SAU) Laboratory Animal Welfare and Ethics guidelines.

### 2.2. Sample Collection for Blood and Tissues

Blood and tissue samples were collected for each of the 9 weeks (from d90 to d153). Nine birds per week were randomly selected, and their blood samples were collected via the wing vein. After being sacrificed via cervical dislocation, six bird livers and ovaries were collected and immediately frozen in liquid nitrogen and subsequently stored at –80 °C for RNA isolation. Body weight (pre-slaughter) and liver and ovary weights were measured too. The liver and ovary samples of three chickens were immersed in a 4% paraformaldehyde solution for histology analysis. 

### 2.3. Histological Staining

The tissues (um/g) were fixed for 24 hours and embedded with paraffin. After hematoxylin and eosin (HE) staining, the tissue sections were used for histological and morphological observation. Moreover, the frozen sections of the liver tissues were carbowax-embedded and stained by oil red O. All the sections were observed with a fluorescence microscope (DP80Digital, Olympus, Tokyo, Japan), and then ten fields were randomly selected for statistical analysis.

### 2.4. Serum Biochemical Parameters Assay

The concentrations of LEC, TG, TC, VTG, VLDLy, estradiol (E_2_), follicle-stimulating hormone (FSH), and luteinizing hormone (LH) in serum were measured by using enzyme-linked immunosorbent assay (ELISA) kits (Baolai Biotechnology Co., Ltd, Yancheng, China), following the manufacturer’s instruction of ELISA kits. 

### 2.5. RNA Isolation and Quantitative Real-Time PCR (qRT-PCR)

Total RNA was isolated from liver and ovary tissues by using Trizol reagent (Takara, Dalian, China) following the manufacturer’s instructions, and the concentration and purity were determined by Nanodrop 2000C (Thermo Fisher Scientific, Waltham, MA, USA) through the A260/280 absorbance ratio. First-strand cDNA was synthesized using PrimeScript RT Reagent Kit (Takara, Dalian, China) according to the manufacturer’s protocols. qRT-PCR was conducted via a CFX96 Real-Time System (Bio-Rad, Hercules, CA, USA) with the following conditions: 95 °C for 3 min; 40 cycles of 95 °C for 10 s and annealing temperature (Table 1) for 20s, followed by a final extension at 72 °C for 20 s, with a melt curve analysis performed at 65~95 °C. The amplification efficiencies of target genes ranged from 95% to 105%. Each qRT-PCR reaction was performed with volumes of 15 μL containing 6.25 μL TB Green^TM^ Premix (Takara), 0.3 μL forward and reverse primers, 1.5 μL cDNA, and 6.65 μL DNase/RNase-Free Deionized Water (Tiangen, Beijing, China). The *GAPDH* was used as the endogenous control to normalize the expression of genes. We choose the d90 group as a reference group (value = 1), and the fold change of gene expressions was quantified using the 2^−ΔΔCt^ method [26], where ΔCt = Ct target gene − Ct was the housekeeping gene, and ΔΔCt = ΔCt − ΔCt was the reference. Gene-specific primers for qRT-PCR were designed using Primer 5 software according to the coding sequence of target genes (Table 1).

### 2.6. Protein Extraction and Western Blot Analysis

Protein extraction from the liver tissue was performed with protein extraction kit (BestBio Biotech Co. Ltd., Shanghai, China), and then the concentration and purity of the protein samples were determined with BCA protein assay kit (BestBio). 

Subsequently, Western blots were performed, as previously described [27], with the following primary and secondary antibodies: rabbit anti-chicken ER-α and ER-β (Abcam, Cambridge, UK; 1:1000), goat anti-rabbit secondary antibodies (Zen-Bio, Chengdu, China), and β-actin (Zen-Bio) was used as a reference. 

### 2.7. Statistical Analysis

The statistical analysis of the data collected was carried out by using SAS 9.3 software (SAS Institute Inc., Cary, NC, USA). All experimental results were presented as the mean ± SE (standard error), and statistical significance was performed with a one-way analysis of variance (ANOVA) followed by the Tukey test [28]. Age was used as a control variable, and the partial correlation coefficients were generated to evaluate the correlation of serum biochemical parameters and gene expression. Values differed significantly at *p* < 0.05.

## 3. Results

### 3.1. Weight Measurements for Body, Liver, and Ovary Tissues

The body, liver, and ovary weights were measured every week from d90 to d153. Our results showed that the body and liver weights increased dramatically from d132, while the ovary weight increased sharply from d139 (Table 2).

### 3.2. Morphological and Histological Analyses

**Figure 1:**
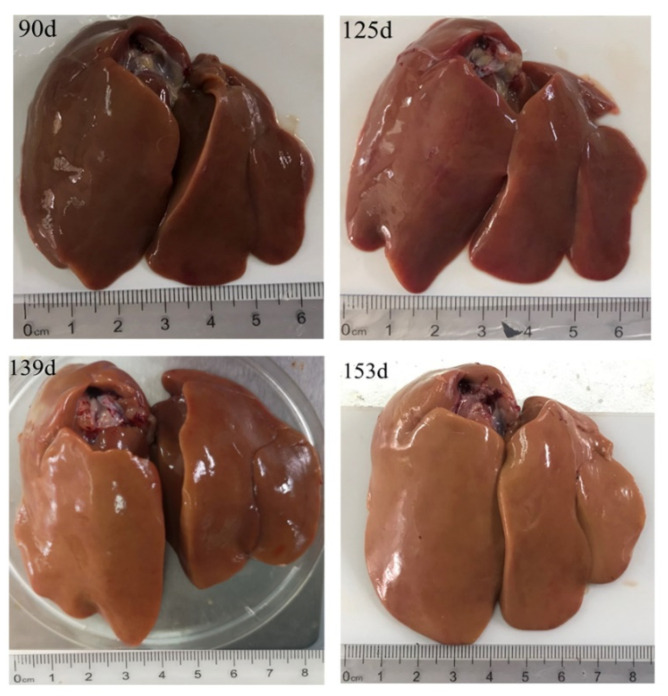
Age-related morphological changes of chicken liver.

**Figure 2:**
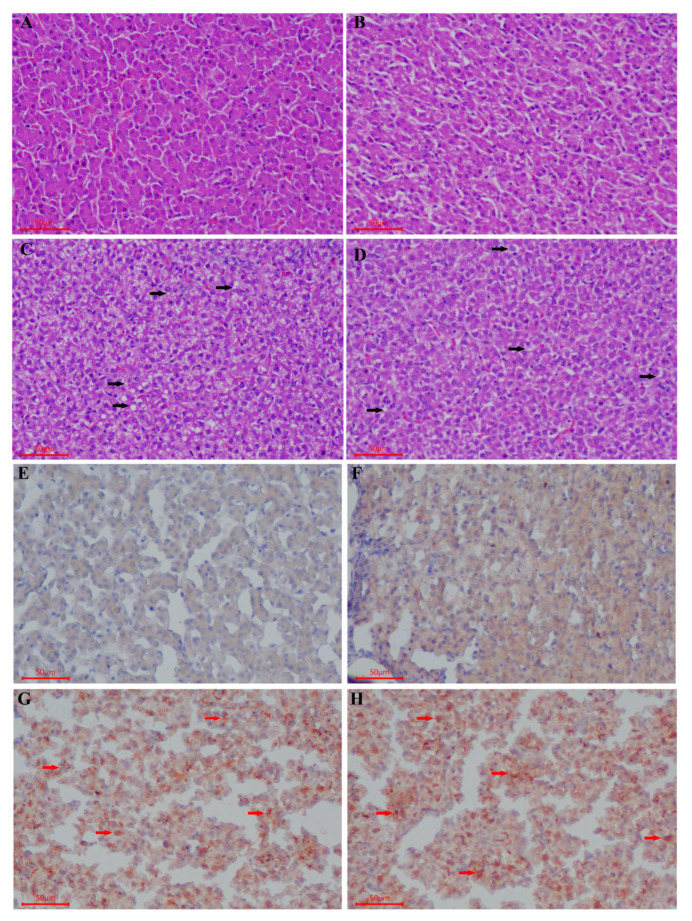
Hematoxylin-eosin (HE) staining of the liver samples with different ages (magnified 200×). (**A**) d90, (**B**) d125, (**C**) d139, and (**D**) d153. Oil red O staining frozen sections of liver samples at different ages (200×). (**E**) d90, (**F**) d125, (**G**) d139, and (**H**) d153. The black arrow indicates the “small fatty vacuoles”, the red arrow indicates the “small lipid droplets”.

**Figure 3:**
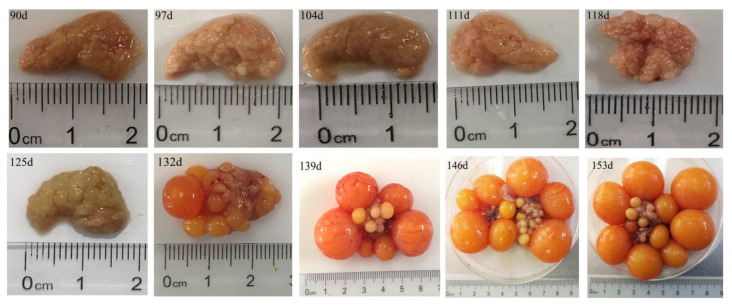
Age-related morphological changes of the chicken ovary.

**Figure 4:**
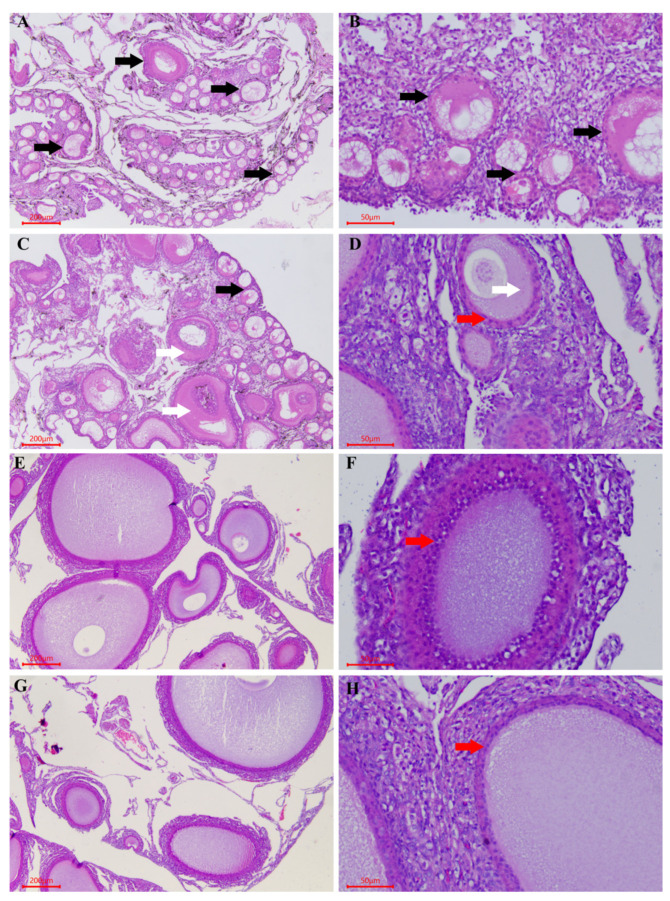
Histological analysis of the chicken ovary at different ages. d90 ((**A**) 40×, (**B**) 200×), d125 ((**C**) 40×, (**D**) 200×), d139 ((**E**) 40×, (**F**) 200×), and d153 ((**G**) 40×, (**H**) 200×). The black arrow indicates the “primordial follicles”, the red arrow indicates the “granulosa cells”, and the white arrow indicates the “lipoproteins”.

We observed in this study that the liver color changed from deep dark to light dark with the increase in age (d125 to d153); this may be due to the intensive yolk precursor synthesis in the liver (Figure 1). Histological analysis showed that the structure of the liver tissue was normal and the hepatocytes were arranged neatly and clearly, while the cytoplasm of the hepatocytes was pink and evenly distributed. Meanwhile, the nucleus had a blue coloration at the center of the cell on d90. We also observed an enlargement in the hepatocytes on d139 and d153, whereas its cytoplasm was loosed and lightly stained, and the presence of a few small fatty vacuoles indicated a mild steatosis (Figure 2A–D). The numerous small lipid droplets appeared in the liver at d125, and then their sizes increased at d139 and d153 with oil red O staining (Figure 2E–H). Additionally, we found that the ovary contained a large number of primordial follicles from d90 to d118, while on d125 the white follicles began to appear. Moreover, on d132 and d139, a very small deposition of yolk was found in one hierarchical follicle (diameter > 12 mm), and five hierarchical follicles were filled with large amounts of egg yolk deposits. D146 was a remarkable growth point when most hens (7 of 9) laid their first eggs with over six yolk-filled follicles in the ovary (Figure 3). Histological analysis showed that the ovary contained a large number of primordial follicles on d90 (Figure 4A,B). Figure 4C,D showed that at d125 the follicles were filled with a small amount of lipoproteins (protein-rich white yolk) which resulted in the appearance of a single layer of granulosa cells of the follicles (Figure 4C,D). Furthermore, at d146 and d153, large yolk deposition in the follicles occurred, resulting in the rapid growth and appearance of more than two layers of granulosa cells in the follicles (Figure 4E–H).

### 3.3. Serum Biochemical Parameter Assay

**Figure 5:**
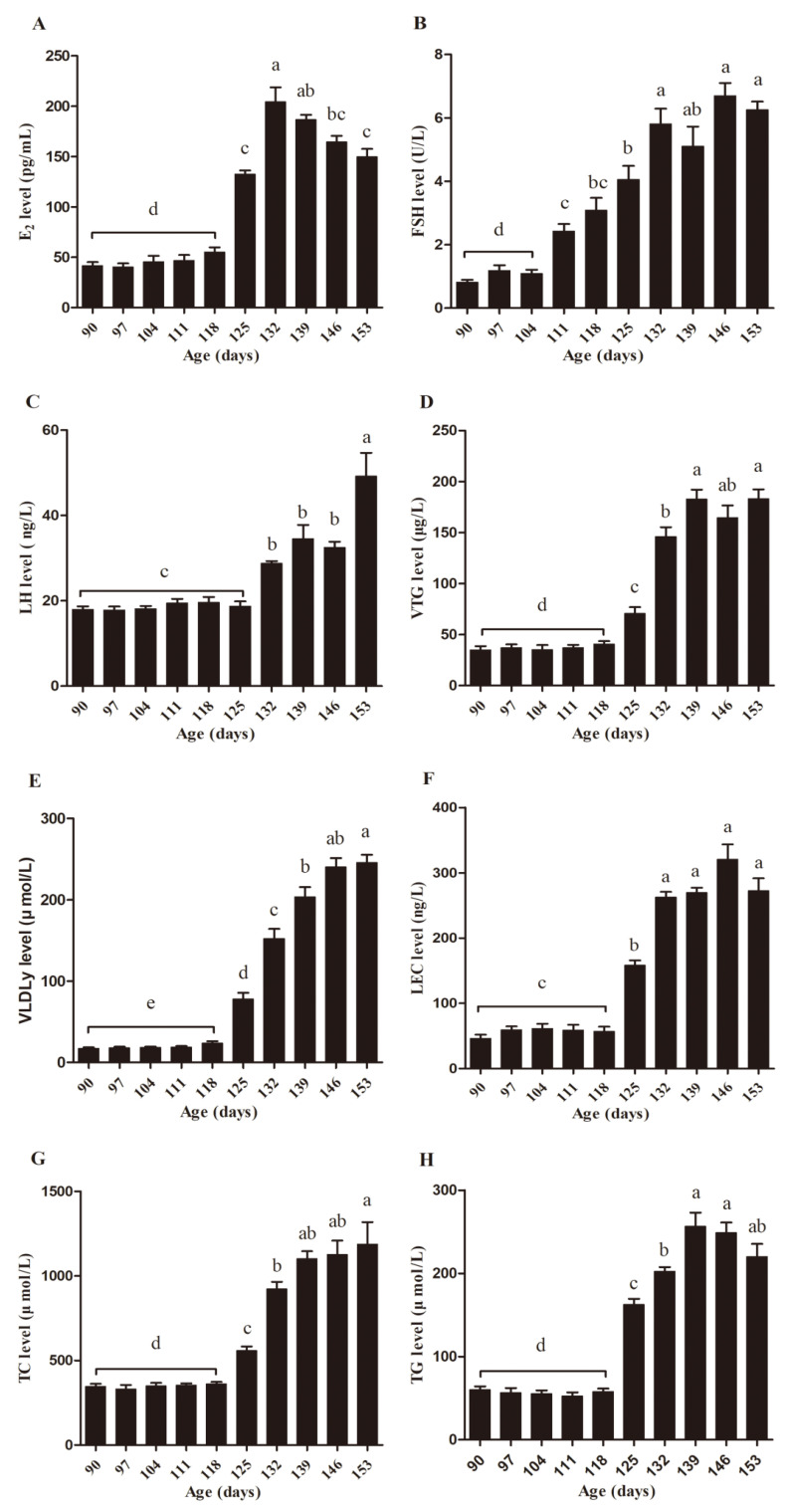
Age-related changes of the biochemical parameters in serum. Levels of estradiol (E_2_) (**A**), follicle-stimulating hormone FSH (**B**), luteinizing hormone (LH) (**C**), vitellogenin (VTG) (**D**), very low density lipoprotein y (VLDLy) (**E**), lecithin (LEC) (**F**), total cholesterol (TC) (**G**), and triglyceride (TG) (**H**) in the blood serum were detected using ELISA kits from d90 to d153. Data was expressed as the mean ± SE (*n* = 9). Different letters indicate significant differences (*p* < 0.05).

The biochemical parameters, including E_2_, FSH, LH, LEC, VTG, VLDLy, TG, and TC in serum were measured (Figure 5). There were no significant differences in the levels of E_2_ from d90 to d118. However, the E_2_ levels increased from d125, reached the peak at d132 (*p* < 0.05), and suddenly decreased significantly from d139 to d153 (*p* < 0.05) (Figure 5A). The FSH levels were not significantly different from d90 to d104, however, they increased significantly from d111 to d132 (*p* < 0.05), and maintained their levels from d132 to d153 (Figure 5B). The level of LH increased significantly from d132 and reached the top on d153 (*p* < 0.05) (Figure 5C). Meanwhile, the levels of LEC, VTG, VLDLy, TG, and TC remained insignificant from d90 to d118 and then increased significantly from d125 (*p* < 0.05) and remained steady from d146 to d153 (Figure 5D–H). Partial correlations show that the age of egg-laying hens was significantly correlated with the levels of E_2_, FSH, LH, LEC, VTG, VLDLy, TG, and TC in serum (Table 3).

### 3.4. Expression Abundance of Hormone Receptors and Yolk Precursor Synthesis-Related Genes

**Figure 6:**
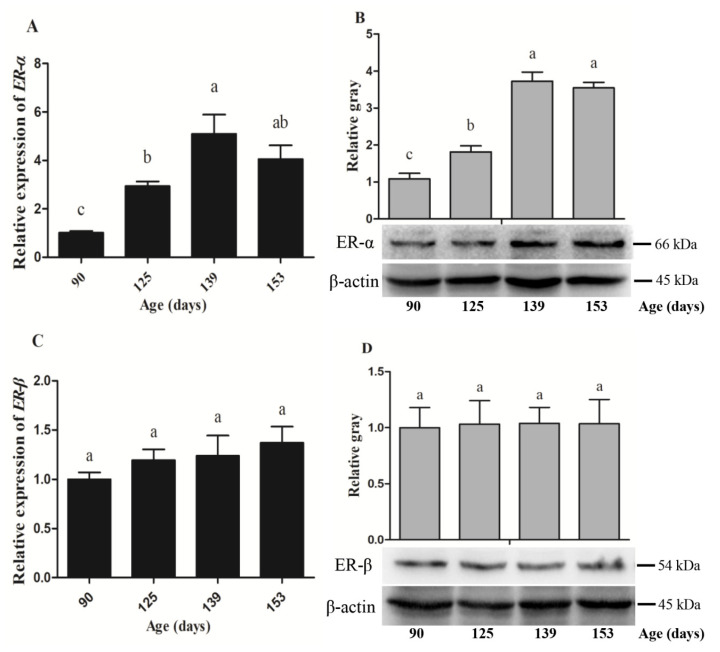
The mRNA and protein expression of ER-α (**A**,**B**) and ER-β (**C**,**D**) in the liver of hens on d90, d125, d139, and d153. Data are expressed as the mean ± SE (*n* = 9). Different letters indicate significant differences (*p* < 0.05).

**Figure 7:**
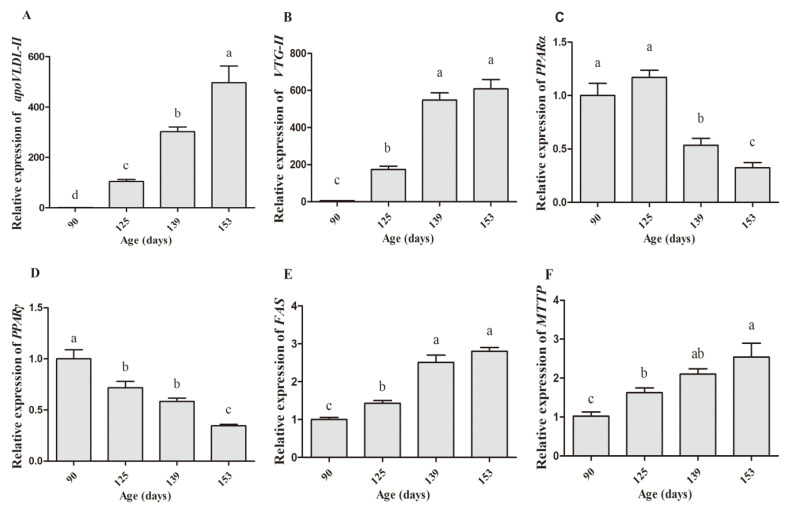
Expression of genes related to yolk precursor formation in the liver of hens on d90, d125, d139, and d153. The mRNA expression of apoVLDL-II (**A**), VTG-II (**B**), PPARα (**C**), PPARγ (**D**), FAS (**E**), and MTTP (**F**) in the liver. Data are expressed as the mean ± SE (*n* = 9). Different letters indicate significant differences (*p* < 0.05).

In this study, the mRNA and protein levels of estrogen receptors and expression patterns of yolk precursor synthesis-related genes were determined on d90, 125, 139, and 153. The results showed that both mRNA and protein levels of estrogen receptor alpha (ER-α) in the liver increased significantly on d125 and reached the peak on d139 (Figure 6A,B). However, there was no significant difference among the time points for the expression abundance of ER-β (Figure 6C,D). In addition, the expression levels of *apoVLDL-II* and *VTG-II* at d90 were almost undetectable, but on d125 they increased remarkably, followed by a rapid rise at d139 and then remained at a higher level at d153. The levels of mRNA expression of peroxisome proliferator-activated α (*PPARα*) and γ (*PPARγ*) showed a gradual downward trend from d90 to d153, meanwhile, fatty acid synthase (*FAS*) and microsomal triglyceride transport protein (*MTTP*) levels presented opposite changing trends (Figure 7). As is shown in Table 4, there were also some significant correlations between age and gene expression. The age of egg-laying hens was positively correlated with the gene expression of ER-α (r = 0.73, *p* < 0.01), apoVLDL-II (r = 0.925, *p* < 0.01), VTG-II (r = 0.889, *p* < 0.01), FAS (r = 0.873, *p* < 0.01), MTTP (r = 0.763, *p* < 0.01) and negatively correlated with PPARα (r = −0.63, *p* < 0.01), and PPARγ (r = −0.808, *p* < 0.01). However, there was no significant correlation between age and ER-β expression.

**Figure 8:**
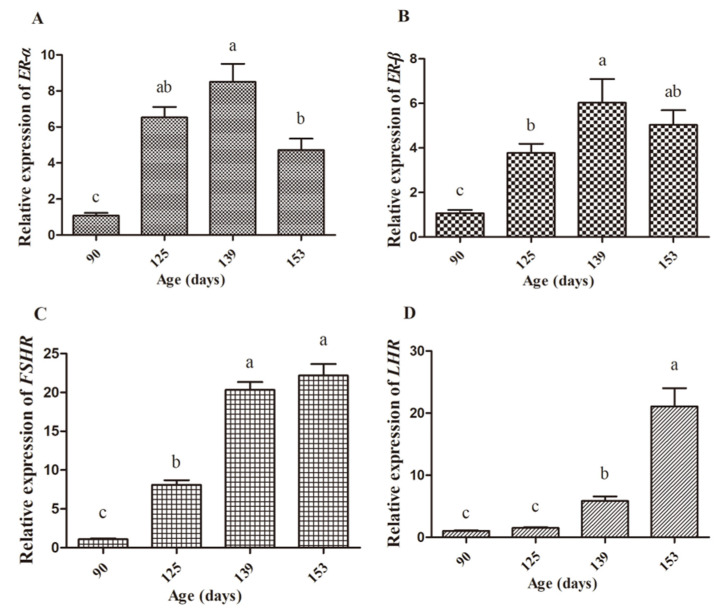
The levels of expression of hormone receptor genes in the ovary of hens on d90, d125, d139, and d153. The mRNA expression of ER-α (**A**), ERβ (**B**), FSHR (**C**), and LHR (**D**) in the ovary. Data are expressed as the mean ± SE (*n* = 9). Different letters indicate significant differences (*p* < 0.05).

In the ovaries, the expression of *ER-α* and *ER-β* reflected a similar pattern from d90 to d153, with a sharp increase from d90 to d139 and then decreased on d153. On d125, *FSHR* expression increased significantly, which was maintained until d139 and then reached its peak at d153. Furthermore, *LHR* was expressed with an increased trend from d139 and reached its top on d153 (Figure 8).

The morphological and histological results demonstrated a change in the liver and the ovary during the period of sexual maturity in hens. With an increase in age, the serum biochemical parameters, expression of yolk precursor synthesis-related genes, and major reproductive hormone receptors increased dramatically. 

## 4. Discussion

Egg laying is one of the normal physiological functions for most oviparous animals after sexual maturity. Egg yolk is part of the fertilized egg that supplies nutrients, energy, and provides immunity for the developing embryo [3,29]. During the laying period of hens, large amounts of the egg yolk proteins and lipids are synthesized in the liver and transported to the ovary [30]. 

Great morphological and histological changes occur in the liver and the ovary of pullet during sexual maturity period. In this study, the weights of the body, liver, and ovary increased dramatically from d139 to d146. Clearly, the liver color was getting brighter with the increasing age (from d90 to d153), which indicated that the vitellogenesis and lipogenesis increased with age. Meanwhile, the ovary also developed some white follicles, hierarchical follicles with obvious egg yolk deposition, and yolk-filled follicles in the hierarchy at d125, d139, and d153, respectively. We speculated that the age-related changes of the weight and the color of the liver were associated with yolk precursor synthesis that occurred in the liver and the deposition of these precursors in the oocyte increased the ovary weight. Further, histological analysis showed that the key period for hepatic lipid synthesis was from d139 to d153. Additionally, the mRNA expression of yolk precursor synthesis-related genes, *apoVLDL-II* and *VTG-II* increased sharply (from almost zero on d90 to hundredfold on d125 and d139) in the liver. The expression of liver lipogenesis-related gene *FAS* and fat transport-related gene *MTTP* also significantly increased on d125 and maintained the level on d139 and d153. However, the expression of lipogenesis-related genes such as *PPARα* and *PPARγ* were significantly decreased on d139 and d153. These results are in accordance with a previous study that showed that the VLDLy levels resisted the lipolytic activity in the laying hen [31]. Accordingly, we found that LEC, VTG, VLDLy, TG, and TC levels in serum increased at d125 and remained steady on d146. This showed that the results obtained for these serum biochemical parameters were consistent with the age-related changes of gene expression in the liver. These findings are in line with the study of Liu et al. (2018), who reported that the aging process influences the capacity of liver yolk precursor synthesis [32].

Previous studies detected that yolk precursor formation in the liver of oviparous animals were largely influenced by estrogens [30,33]. E_2_ activates the expression of liver yolk precursor synthesis-related genes by binding to their specific estrogen receptors, especially *ERα* [34,35]. Moreover, previous studies demonstrated that exogenous estrogen administration to hens stimulated the synthesis of hepatic apoB and VLDL [36,37] and induced hepatic biosynthesis of phospholipid and triacylglycerol in birds [38]. In this study, the E_2_ levels in the serum significantly increased at d125, this development was similar to a previous report that found that the E_2_ levels in hen plasma at d119 were higher than d91 [39]. On d125, there was a rapid rise in the expression of *ER-α*; while the expression of *apoVLDL-II* and *VTG-II* levels in the liver also increased significantly on d125 and 139, however, *ER-β* expression recorded no significant difference. These results are consistent with previous researches that reported that estrogen released in hens during the laying period predominantly increased the mRNA expression of *ApoVLDL-II* and *VTG-II* by acting on *ER-α* [35,40].

In addition, the rapid follicle development in the ovary of chickens is accompanied with absorption and deposition of yolk precursors. Follicular growth and development are regulated by FSH and LH [41]. In the process of follicular growth and maturation, FSH secreted by the pituitary gland plays a leading role by stimulating the follicle to secrete estrogen through the FSH receptor of the ovary, promoting the deposition of yolk material and enables the follicle to reach maturity as soon as possible [42]. When follicular growth and development reach a certain stage, FSH can promote the granulosa cells with lumen follicles to generate LH receptors and induce LH secretion [43]. In the present study, we found that the FSH levels in serum increased from d111, which happened earlier before the increase for the E_2_ level. This indicated that a rise in the level of E_2_ may depend directly on the increase of FSH. We further detected the mRNA expression of *ER-α*, *ER-β*, *FSHR*, and *LHR* in the ovaries and the results showed increased expressions for *FSHR*, *LHR*, *ER-α,* and *ER-β* on d139, which indicated that FSH, LH, and E_2_ jointly regulated follicular development during sexual maturity in hens.

## 5. Conclusions

Generally, the correlation between liver-blood-ovary alliances regulated the transport and exchange of synthetic substances to ensure synchronous development and functional coordination between the liver and ovary. Moreover, the age-related hormone changes directly influenced the transcriptional changes of genes related to fat metabolism and yolk precursor synthesis. We confirm that E_2_ is an activator that is regulated by FSH, which induces histological and functional changes in hepatocytes through the ER-α pathway.

## Figures and Tables

**Table 1:** Primers used for quantitative real-time PCR (qRT-PCR).

Gene	Sequence (5’-3’)	Product Length (bp)	Annealing Temperature (°C)	Accession Number
*ER-α*	F: TGTGCTGTGTGCAACGACTA	167	57	NM_205183.2
	R: CAGGCCTGGCAACTCTTTCT			
*ER-β*	F: GGCTGCAACCCGTGTAAAAG	189	58	NM_204794.2
	R: GCCCAGCCAATCATGTGAAC			
*apoVLDL-II*	F: CCTTAGCACCACTGTCCCTG	130	58	NM_205483.2
	R: AGCTCTAGGGGACACCTTGT			
*VTG-II*	F: AACTACTCGATGCCCGCAAA	179	58	NM_001031276.1
	R: ACCAGCAGTTTCACCTGTCC			
*FAS*	F: TGCTATGCTTGCCAACAGGA	128	59	NM_205155.3
	R: ACTGTCCGTGACGAATTGCT			
*PPARα*	F: AGGCCAAGTTGAAAGCAGAA	155	60	NM_001001464.1
	R: TTTCCCTGCAAGGATGACTC			
*PPARγ*	F: TGACAGGAAAGACGACAGACA	164	59	NM_001001460.1
	R: CTCCACAGAGCGAAACTGAC			
*MTTP*	F: GTTCTGAAGGACATGCGTGC	120	58	NM_001109784.2
	R: GATGTCTAGGCCGTACGTGG			
*GAPDH*	F: TCCTCCACCTTTGATGCG	144	60	NM_204305.1
	R: GTGCCTGGCTCACTCCTT			

*ER-α/β:* estrogen receptor (ER)-alpha/beta; *ApoVLDL-II*: very low density apolipoprotein-II; *VTG-II*: vitellogenin-II; *FAS*: fatty acid synthase; *PPARα/γ*: peroxisome proliferator-activated receptor-alpha/gamma; *MTTP*: microsomal triglyceride transport protein; F: Forward primer; R: Reverse primer.

**Table 2:** Weight measurements from d90 to d153.

Age (d)	Body Weight (g)	Liver Weight (g)	Ovary Weight (g)	Follicle Numbers (Diameter > 12 mm)
90	1085.33 ± 71.39 ^b^	21.46 ± 0. 60 ^b^	0.45 ± 0.03 ^c^	-
97	1136.20 ± 24.44 ^b^	22.52 ± 2.59 ^b^	0.47 ± 0.01 ^c^	-
104	1084.97 ± 48.56 ^b^	22.21 ± 2.76 ^b^	0.48 ± 0.01 ^c^	-
111	1127.59 ± 142.19 ^b^	23.30 ± 4.46 ^b^	0.52 ± 0.04 ^c^	-
118	1127.59 ± 142.19 ^b^	24.79 ± 4.39 ^b^	0.62 ± 0.12 ^c^	-
125	1338.51 ± 138.49 ^b^	22.92 ± 3.29 ^b^	0.74 ± 0.17 ^c^	-
132	1713.40 ± 131.03 ^a^	39.82 ± 7.78 ^a^	1.50 ± 0.17 ^c^	1.00 ± 0.23 ^c^
139	1760.00 ± 101.76 ^a^	37.13 ± 7.73 ^a^	17.80 ± 10.96 ^b^	4.44 ± 0.28 ^b^
146	1731.12 ± 176.08 ^a^	36.69 ± 6.83 ^a^	36.47 ± 5.41 ^a^	6.00 ± 0.24 ^a^
153	1794.00 ± 228.54 ^a^	36.64 ± 4.99 ^a^	47.68 ± 13.64 ^a^	6.44 ± 0.17 ^a^

Data are shown as the mean ± standard error (SE) (*n* = 9). Means within a column marked without the same superscripts differed significantly (*p* < 0.05).

**Table 3:** Partial correlation coefficients between age and serum biochemical parameters.

Variable	E_2_	FSH	LH	VTG	VLDLy	LEC	TC	TG
Age	0.824 **	0.874 **	0.765 **	0.867 **	0.913 **	0.872 **	0.861 **	0.863 **

The superscript double asterisk (**) denotes statistical difference of *p* < 0.01.

**Table 4:** Partial correlation coefficients between age and gene expression.

Variable	ER-α	ER-β	apoVLDL-II	VTG-II	FAS	PPARα	PPARγ	MTTP
Age	0.73 **	0.319	0.925 **	0.889 **	0.873 **	−0.63 **	−0.808 **	0.763 **

The superscript double asterisk (**) denotes a statistical difference of *p* < 0.01.
